# Diagnostic Challenges in Differentiating Unicystic Ameloblastoma From Odontogenic Cysts: Report of Two Cases

**DOI:** 10.1155/crid/2944809

**Published:** 2026-05-18

**Authors:** Chaima Khalifa, Maroua Garma, Afef Slim, Adel Bouguezzi, Sameh Sioud, Hajer Hentati

**Affiliations:** ^1^ Department of Oral Medicine and Oral Surgery, Monastir, Tunisia; ^2^ Laboratory of Oral Health and Maxillofacial Rehabilitation (LR12ES11), Faculty of Dental Medicine of Monastir, University of Monastir, Monastir, Tunisia, fmdm.rnu.tn

**Keywords:** cone beam computed tomography, differential diagnosis, histopathology, jaw tumors, odontogenic cyst, unicystic ameloblastoma

## Abstract

Ameloblastomas are benign odontogenic tumors characterized by local aggressiveness and a high potential for recurrence. Unicystic ameloblastoma (UA) is an uncommon variant that often mimics odontogenic cysts, making preoperative diagnosis challenging. Early and accurate differentiation is essential for guiding appropriate treatment planning and minimizing the risk of recurrence. This study is aimed at reporting two cases highlighting the diagnostic dilemma posed by UA. The first case involves a 65‐year‐old male patient with a well‐defined unilocular radiolucency extending from Teeth 35 to 44. Clinical and radiographic findings initially suggested a benign odontogenic cyst. However, cone beam computed tomography (CBCT) revealed cortical perforation, and histopathological examination confirmed a diagnosis of a mural unicystic ameloblastoma. The second case concerns a 20‐year‐old female patient who consulted with the chief complain of incidental discovery of a radiolucent image associated with an impacted wisdom mandibular right tooth during follow‐up orthodontic treatment on panoramic radiography. CBCT showed cortical expansion and thinning, with mandibular canal displacement. The differential diagnosis included a dentigerous cyst, an odontogenic keratocyst, and UA. Histopathological analysis confirmed a follicular ameloblastoma. These cases emphasize the importance of correlating clinical, radiographic, and histopathological findings to differentiate UA from cystic odontogenic lesions. Advanced imaging modalities such as CBCT, combined with histological examination, remain essential for a precise diagnosis and suitable treatment plan.

## 1. Introduction

Ameloblastomas are defined as benign epithelial intraosseous odontogenic tumors arising from enamel, dental follicles, periodontal ligaments, or the linings of odontogenic cysts, marked by their progressive growth, potential to destroy adjacent tissues, and propensity for local recurrence if not completely excised. The exact cause of ameloblastoma is unknown, but factors such as localized trauma, nutritional deficiencies, inflammation, genetic mutations, and molecular pathway abnormalities may play a role, potentially contributing to its aggressiveness and metastatic potential [1]

According to the World Health Organization 2022, ameloblastomas are benign epithelial odontogenic tumors divided into five subtypes: unicystic, extraosseous, conventional, adenoid, and metastasizing ameloblastoma [2]. Unicystic ameloblastoma (UA) is a rare variant, accounting for approximately 10%–15% of all intraosseous ameloblastomas [3]. It typically presents as a unilocular radiolucent lesion, often mimicking more common odontogenic cysts such as dentigerous or radicular cysts, particularly in younger patients [4]. This overlapping clinical and radiographic presentation poses a significant diagnostic challenge, frequently leading to initial misclassification and inappropriate management [5]. Despite its seemingly benign cystic appearance, UA harbors a neoplastic potential that requires a distinctly different therapeutic approach than nonneoplastic lesions. The definitive diagnosis relies heavily on histopathological examination. Early and accurate identification of UA is crucial to guide adequate treatment and reduce the recurrence rate [6].

This study is aimed to report and discuss two clinical cases of UA involving two patients of different age ranges in order to highlight the diagnostic difficulties associated with UA, discuss key distinguishing features from odontogenic cysts, and propose a more systematic approach to succeed in differential diagnosis in routine clinical practice.

## 2. Case Report 1

A 65‐year‐old male patient, noninsulin‐dependent, balanced diabetic and hypertensive was referred to the academic dental clinic of Monastir, Tunisia, for the discovery of a radiolucent lesion in the anterior mandible. The extraoral examination was normal. Intraorally, poor oral hygiene was noted. Vitality tests were positive for all teeth in the mandibular anterior region, except for Teeth 41 and 42. The corresponding teeth show no signs of discoloration and no history of trauma. The panoramic radiograph revealed a well‐defined multilocular radiolucent lesion extending from Tooth 35 to 44, surrounded by a thin sclerotic margin (Figure 1).

**Figure 1:**
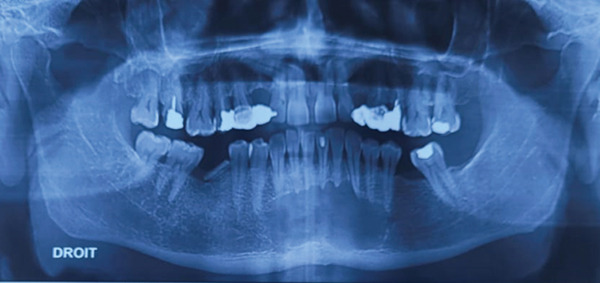
Panoramic radiograph showing a well‐defined radiolucent lesion with multilocular borders extending from Tooth 35 to 44, surrounded by a thin sclerotic margin.

The lesion appeared in close contact with the incisor region. Given this radiographic appearance, a 3D imaging examination was requested. The CBCT showed a well‐circumscribed osteolytic lesion with cortical perforation in some areas (Figure 2). The measure of the lesion is 4.4 cm. The differential diagnoses included an inflammatory cyst, an odontogenic keratocyst, a simple bone cyst, and ameloblastoma. The patient underwent an endodontic treatment of Teeth 41 and 42, followed by surgical enucleation of the lesion (Figure 3). The specimen was sent for histopathological examination, which revealed a mural variant of UA. A rigorous follow‐up protocol was established. The panoramic radiograph at 6 months showed ossification of the lesion with no signs of recurrence (Figure 4).

**Figure 2:**
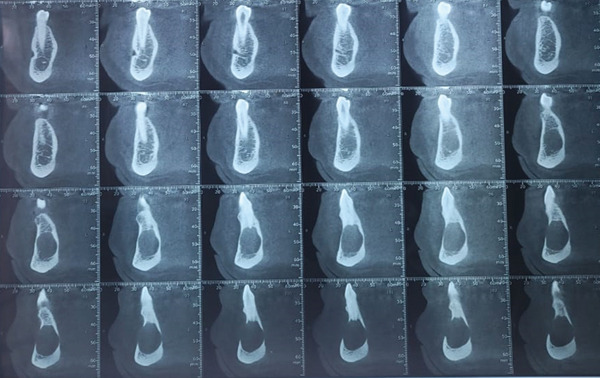
Coronal view of CBCT: a well‐circumscribed osteolytic lesion with cortical perforation in some areas.

**Figure 3:**
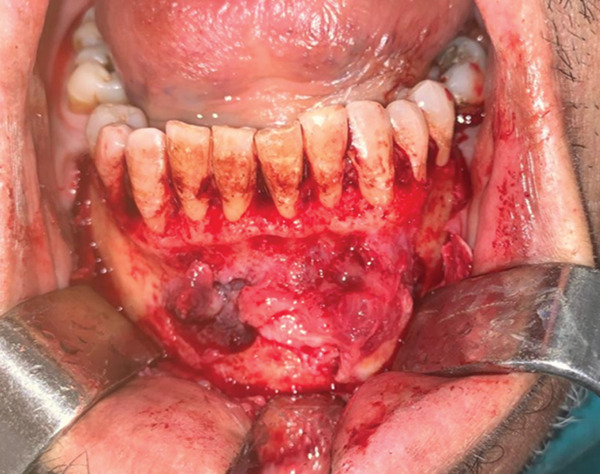
Intraoperative view of the lesion enucleation.

**Figure 4:**
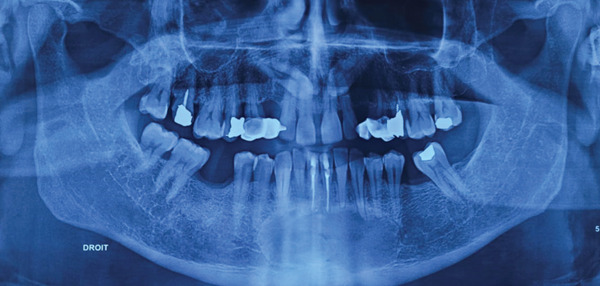
Panoramic radiography after 6 months follow‐up postoperative period.

## 3. Case Report 2

A 20‐year‐old female patient presented to the Department of Oral Medicine and Oral Surgery at the Academic Dental Clinic of Monastir, Tunisia, with the chief complaint of incidental discovery of a radiolucent image associated with the impacted right mandibular wisdom tooth during follow‐up orthodontic treatment on panoramic radiography. Her familial and past medical history were noncontributory. The clinical examination was unremarkable. However, radiographic investigation through an orthopantomogram showed a well‐defined radiolucent image surrounded by a thin sclerotic border, encompassing the crown of the impacted Tooth 48 (Figure 5).

**Figure 5:**
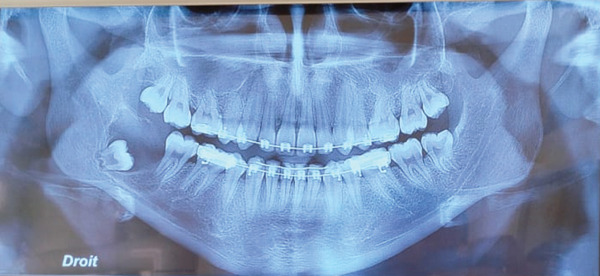
Orthopantomogram showing a well‐demarcated radiolucent lesion surrounded by a thin sclerotic border, encompassing the crown of an impacted Tooth 48.

A CBCT scan was requested, showing a radiolucent lesion surrounding the cervical area of Tooth 48, with buccal expansion and areas of lingual cortical perforation. The lesion measures 2.2 cm. The mandibular canal appeared displaced (Figure 6).

**Figure 6:**
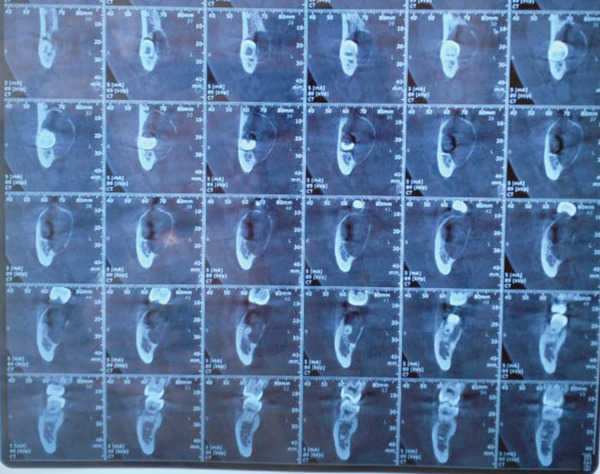
Coronal view of CBCT: A radiolucent lesion encircling the cervical area of Tooth 48, with buccal expansion and areas of lingual cortical perforation. The mandibular canal appeared displaced.

Based on the clinical and radiological findings, the differential diagnoses included a dentigerous cyst, an odontogenic keratocyst, and an ameloblastoma. Surgical management involved the extraction of the impacted tooth along with lesion enucleation (Figure 7). The specimen was sent for histopathological examination, which confirmed the diagnosis of a follicular ameloblastoma, which is a solid variant. This diagnosis diverged from the initial radiographic finding of a unicystic lesion and reflects a different histological type rather than a misdiagnosis or progression. The patient has continued to undergo regular clinical and radiological follow‐up, which has been uneventful to date (Figure 8).

**Figure 7:**
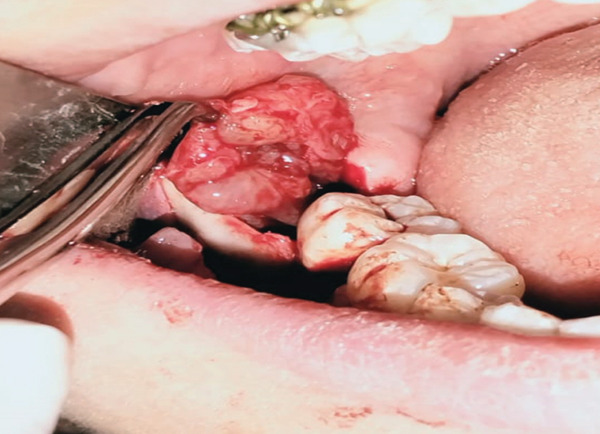
Intraoperative view during lesion enucleation.

**Figure 8:**
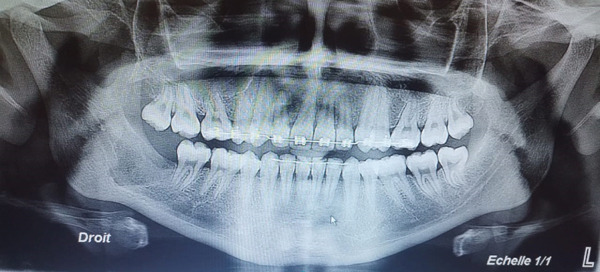
Panoramic radiograph after 6 months showing an ossification of the lesion.

In both cases, radiographic assessment was crucial in narrowing the differential diagnosis, as the lesions initially mimicked common odontogenic cysts. Although unicystic ameloblastoma typically appears as a unilocular radiolucency resembling dentigerous or radicular cysts, subtle features such as cortical expansion, scalloped margins, and root resorption favored a neoplastic process. Differentiation from odontogenic cysts, which usually show minimal expansion and rare resorption, and from solid ameloblastoma variants with multilocular “soap‐bubble” patterns remains challenging. Thus, although imaging provides important diagnostic clues, definitive diagnosis ultimately requires histopathological confirmation.

## 4. Discussion

The accurate diagnosis of UA remains a persistent challenge in oral pathology due to its deceptively benign clinical and radiographic features. Frequently presenting as a well‐defined, unilocular radiolucency, often associated with the crown of an unerupted tooth. UA is commonly misdiagnosed as odontogenic cysts such as dentigerous or radicular cysts [7]. This diagnostic overlap is particularly pronounced in younger patients, where both entities commonly occur [3].

UA typically presents as a painless, slow‐growing swelling in the jaw, most commonly affecting the posterior mandible of young adults [8]. Clinically, it may cause facial asymmetry, cortical bone expansion, and occasionally tooth displacement or root resorption. Despite its often‐innocuous presentation, UA can exhibit locally aggressive behavior. Due to its cyst‐like appearance, it is frequently misdiagnosed as an odontogenic cyst [8]. Radiographically, the unilocular appearance of UA lacks the typical multilocular “soap bubble” or “honeycomb” pattern seen in conventional ameloblastomas [9]. This further contributes to diagnostic confusion, especially when the lesion is asymptomatic and discovered incidentally. Although imaging modalities like cone‐beam CT may reveal subtle differences such as cortical bone expansion or thinning, these findings are not pathognomonic. Consequently, reliance on imaging alone can lead to misdiagnosis and inappropriate treatment planning [10, 11].

To better guide the differential diagnosis, it is essential to identify key clinical and radiological distinctions between odontogenic cysts and UAs.

Clinically, odontogenic cysts are typically asymptomatic, slow growing, and rarely cause significant cortical perforation or tooth root resorption. In contrast, UA may present with painless swelling but more frequently causes cortical expansion, tooth displacement, or resorption, especially in mural variants [12].

Radiographically, odontogenic cysts such as dentigerous or radicular cysts typically present as pericoronal, unilocular radiolucencies with smooth corticated margins and minimal effects on surrounding structures. In contrast, UA may mimic this appearance but often exhibits subtle signs of aggressiveness, including scalloped or irregular borders, cortical thinning or rupture, and displacement or resorption of adjacent teeth. Odontogenic keratocysts can also resemble UA but generally extend along the bone with minimal buccolingual expansion and rarely cause root resorption. CBCT is particularly valuable in detecting these differences, especially in assessing cortical integrity and the relationship to adjacent anatomical structures such as the mandibular canal or mental foramen [10]. Therefore, variations in outline, locularity, and impact on neighboring structures are critical in refining the radiographic differential diagnosis, though definitive confirmation ultimately requires histopathological examination.

Histopathology remains the gold standard for definitive diagnosis. Nevertheless, even this can be complex. UA exists in several histologic subtypes: luminal, intraluminal, and mural, each with distinct biological behaviors and therapeutic implications [2]. Particularly, the mural variant demonstrates invasion into the cyst wall and is associated with a higher risk of recurrence, often necessitating more aggressive treatment such as resection rather than simple enucleation. Failure to identify these microscopic features may lead to incomplete treatment and long‐term complications. One of the main limitations in routine diagnosis is the sampling error during biopsy. Incisional biopsies may not capture the ameloblastic component if limited to a mural or intraluminal proliferation [13]. Therefore, thorough histological examination of the entire specimen, often after complete enucleation, is essential for an accurate diagnosis. In some cases, multiple sections and immunohistochemical staining may be required to differentiate UA from cystic lesions exhibiting hyperplastic epithelial lining or reactive changes [14]. From a clinical standpoint, a high rate of suspicion is necessary when evaluating cystic lesions of the jaws, especially in younger patients or when radiographic findings are atypical. Characteristics such as root resorption, significant cortical expansion, or failure of a lesion to respond to conventional cyst treatment should prompt further investigation. Moreover, long‐term follow‐up is mandatory given the potential for recurrence, particularly in cases treated conservatively [15]. In summary, distinguishing UA from odontogenic cysts demands a multidisciplinary approach involving careful clinical evaluation, advanced imaging, and, most importantly, meticulous histopathological analysis. A standardized diagnostic algorithm could aid clinicians in minimizing misdiagnosis and optimizing treatment outcomes [16].

## 5. Take‐Home Message

Radiographic features can provide valuable clues in differentiating UA from other odontogenic lesions, but significant overlap exists with dentigerous cysts and keratocysts. Key radiographic indicators, such as pericoronal attachment beyond the CEJ, scalloped margins, buccolingual expansion, and root resorption, should raise suspicion for UA. Clinicians should maintain a high index of suspicion and consider histopathological examination as the definitive diagnostic tool. Early identification and accurate differentiation are crucial for appropriate surgical planning and minimizing recurrence risk. Cross‐sectional imaging, such as CBCT, can aid in assessing cortical integrity and lesion extent.

## 6. Conclusion

UA poses a significant diagnostic challenge due to its clinical and radiographic resemblance to odontogenic cysts. The two cases presented highlight the importance of thorough diagnostic assessment, including advanced imaging and histopathological examination, to ensure accurate diagnosis and appropriate treatment. Early and correct identification of UA is essential to avoid inappropriate treatment, reduce the risk of recurrence, and improve patient outcomes. A systematic, multidisciplinary approach remains vital in differentiating UA from other cystic jaw lesions in clinical practice.

## Author Contributions

Chaima Khalifa: conceptualization, methodology, investigation, data curation, writing—original draft, writing—review and editing. Maroua Garma: investigation, data curation, writing—original draft. Afef Slim: methodology, validation, writing—review and editing. Adel Bouguezzi: supervision, validation, writing—review and editing. Sameh Sioud: resources, data curation, visualization. Hajer Hentati: supervision, project administration, writing—review and editing.

## Funding

No funding was received for this manuscript.

## Disclosure

All authors have read and approved the final version of the manuscript. Khalifa Chaima had full access to all of the data in this study and takes complete responsibility for the integrity of the data and the accuracy of the data analysis.

## Ethics Statement

Written informed consent was obtained from the patients for publication of their clinical information and accompanying images. Ethical approval was not required for this case report in accordance with institutional and national guidelines.

## Conflicts of Interest

The authors declare no conflicts of interest.

## Data Availability

The authors confirm that the data supporting the findings of this study are available within the article and/or its supporting information.
